# The HDAC inhibitor SB939 overcomes resistance to BCR-ABL kinase Inhibitors conferred by the *BIM* deletion polymorphism in chronic myeloid leukemia

**DOI:** 10.1371/journal.pone.0174107

**Published:** 2017-03-16

**Authors:** Muhammad Rauzan, Charles T. H. Chuah, Tun Kiat Ko, S. Tiong Ong

**Affiliations:** 1 Cancer and Stem Cell Biology Signature Research Programme, Duke-NUS Medical School, Singapore; 2 Department of Haematology, Singapore General Hospital, Singapore; 3 Department of Medical Oncology, National Cancer Centre Singapore, Singapore; 4 Department of Medicine, Duke University Medical Center, Durham, North Carolina, United States of America; Istituto di Genetica Molecolare, ITALY

## Abstract

Chronic myeloid leukemia (CML) treatment has been improved by tyrosine kinase inhibitors (TKIs) such as imatinib mesylate (IM) but various factors can cause TKI resistance in patients with CML. One factor which contributes to TKI resistance is a germline intronic deletion polymorphism in the *BCL2-like 11* (*BIM*) gene which impairs the expression of pro-apoptotic splice isoforms of *BIM*. SB939 (pracinostat) is a hydroxamic acid based HDAC inhibitor with favorable pharmacokinetic, physicochemical and pharmaceutical properties, and we investigated if this drug could overcome *BIM* deletion polymorphism-induced TKI resistance. We found that SB939 corrects *BIM* pre-mRNA splicing in CML cells with the *BIM* deletion polymorphism, and induces apoptotic cell death in CML cell lines and primary cells with the *BIM* deletion polymorphism. More importantly, SB939 both decreases the viability of CML cell lines and primary CML progenitors with the *BIM* deletion and restores TKI-sensitivity. Our results demonstrate that SB939 overcomes *BIM* deletion polymorphism-induced TKI resistance, and suggest that SB939 may be useful in treating CML patients with *BIM* deletion-associated TKI resistance.

## Introduction

Chronic myeloid leukemia (CML) is a disease defined by the presence of the BCR-ABL fusion protein, a constitutively active kinase produced by the 9,22 translocation which is sufficient to transform hematopoietic cells [1]. ABL-specific tyrosine kinase inhibitors (TKIs), such as imatinib mesylate (IM), have significantly improved CML treatment, and prevent transformation to the deadly blast phase of the disease [2, 3]. However, patients with suboptimal TKI responses are at risk of developing TKI-resistance and progressing to blast phase [4]. We previously described a germline intronic deletion polymorphism in the *BCL2-like 11* (*BIM*) gene that was sufficient to mediate TKI resistance in CML [5]. In cells which harbor the *BIM* deletion polymorphism, splicing of *BIM* pre-mRNA is biased toward the inclusion of exon 3 (E3) and exclusion of exon 4 (E4). Since the pro-apoptotic BH3 domain is encoded by E4, the deletion promotes the expression of non-apoptotic BIM isoforms (which retain E3 and encode the non-functional BIMγ protein) over pro-apoptotic isoforms (which exclude 3 and include E4, and encode the pro-apoptotic BIMEL, BIML, and BIMS proteins), thereby impairing the pro-apoptotic TKI response and confering partial TKI-resistance [5].

Cancer cells usually have aberrant histone acetylation profiles and it was reported that histone deacetylases (HDACs) activities are essential in establishing a tumor phenotype [6]. Additionally, non-histone proteins such as p53, Hsp90 and Ku70 had aberrant acetylation in IM-resistant CML cell lines due to the down-regulation of histone acetyltransferase (HAT) and upregulation of HDACs [7]. HDAC inhibitors (HDACi) change the acetylation status of both histone- and non-histone proteins, hence altering cell proliferation, transcriptional regulation, and other cellular functions of cancer cells [8]. Vorinostat, an FDA-approved HDACi, was effective in overcoming *BIM* deletion polymorphism-induced TKI resistance in non-small-cell lung cancer (NSCLC) when combined with an EGFR TKI [9]. However, a newer HDACi, SB939 (pracinostat), was found to have better pharmacokinetic, physicochemical, and pharmaceutical properties than vorinostat and is currently in phase II clinical trials for a number of cancers [10]. Additionally, SB939 was shown to overcome TKI resistance in T315I mutants of BCR-ABL when co-treated with an aurora kinase inhibitor [11]. In this study, we investigated the efficacy of SB939, alone or with IM, in overcoming TKI resistance mediated by *BIM* polymorphism in CML. Our results indicate that SB939 does enhance IM lethality in CML cells, including those with the *BIM* deletion polymorphism.

## Material and methods

### Cell lines, culture and chemicals

The CML cell line K562 was purchased from ATCC. Genome–edited K562 cells with or without the *BIM* deletion polymorphism were generated as previously described [5]. Imatinib and SB939 were purchased form SelleckChem (USA). These drugs were dissolved in DMSO and stored at -20°C.

### Real-time quantitative PCR (qPCR) analysis of exon-specific *BIM* transcripts

Total cellular RNA were extracted using RNeasy Mini Kit (Qiagen, Germany). Superscript III First-strand Synthesis System (Invitrogen, USA) were used to reverse transcribe the RNA which is then quantitatively analysed using the iQ5 Multicolor Real-Time Detection System (Bio-Rad, USA) with a total reaction volume of 25 ul. Primers were annealed at 59°C for 20 s, and the amplicon was extended at 72°C for 30 s. The total number of cycles was 40. The following primers were used: *BIM* exon 3 (forward: 5′-CAATGGTAGTCATCCTAGAGG-3′; reverse: 5′-GACAAAATGCTCAAGGAAGAGG-3′), *BIM* exon 4 (forward: 5′-TTCCATGAGGCAGGCTGAAC-3′; reverse: 5′-CCTCCTTGCATAGTAAGCGTT-3′).

### Immunoblot and antibodies

We used the following antibodies for immunoblotting: BCR-ABL (#2862), p-BCR-ABL (#2861), BIM (#2819), CASPASE 3 (#9662), STAT5 (#9310), p-STAT5 (#9359), PARP (#9542) (all from Cell Signaling Technology, USA) and beta-ACTIN (#AC-15, Sigma, USA). The antibody dilutions used were 1 in 1,000, except for PARP (1 in 2,000), BIM (1 in 500) and β-ACTIN (1 in 5,000). HRP-conjugated secondary antibodies were specific to rabbit (Sigma, USA) or mouse IgG (Santa Cruz biotechnology, USA). The protein bands on the membrane were visualized using the Western Lightning chemiluminescence reagent (PerkinElmer, USA).

### Apoptosis assay

Apoptosis in CML cell lines and primary CML samples were measured by flow cytometry with the Annexin V-FITC antibody (#640906, Biolegend, USA) and DAPI (Sigma-Aldrich, USA). Ten thousand events were recorded and the gated region and data were analysed using FlowJo software (Treestar, USA).

### Ethics committee approval

Clinical CML samples were obtained from the Singapore General Hospital. Written informed consent and institutional review board approval were obtained from the relevant individuals and SingHealth Centralised Institutional Review Board (CIRB) respectively.

### Drug treatment for primary CML cells

Primary peripheral blood mononuclear cells (PBMCs) were subjected to drug treatment for 72 hours in StemPro (with supplements as indicated in [12]; Life Technologies, USA).

### Colony formation assay (CFA)

Primary cells and cell lines were seeded with 500–2000 cells per 35mm plate (in duplicates), in drug-free methylcellulose (H4434; Stemcell Technologies, Canada). Colonies were counted after 10–14 days post-seeding.

## Results

### SB939 corrects *BIM* splicing and induces apoptotic cell death in *BIM* deletion polymorphism-containing CML cells

**Fig 1:**
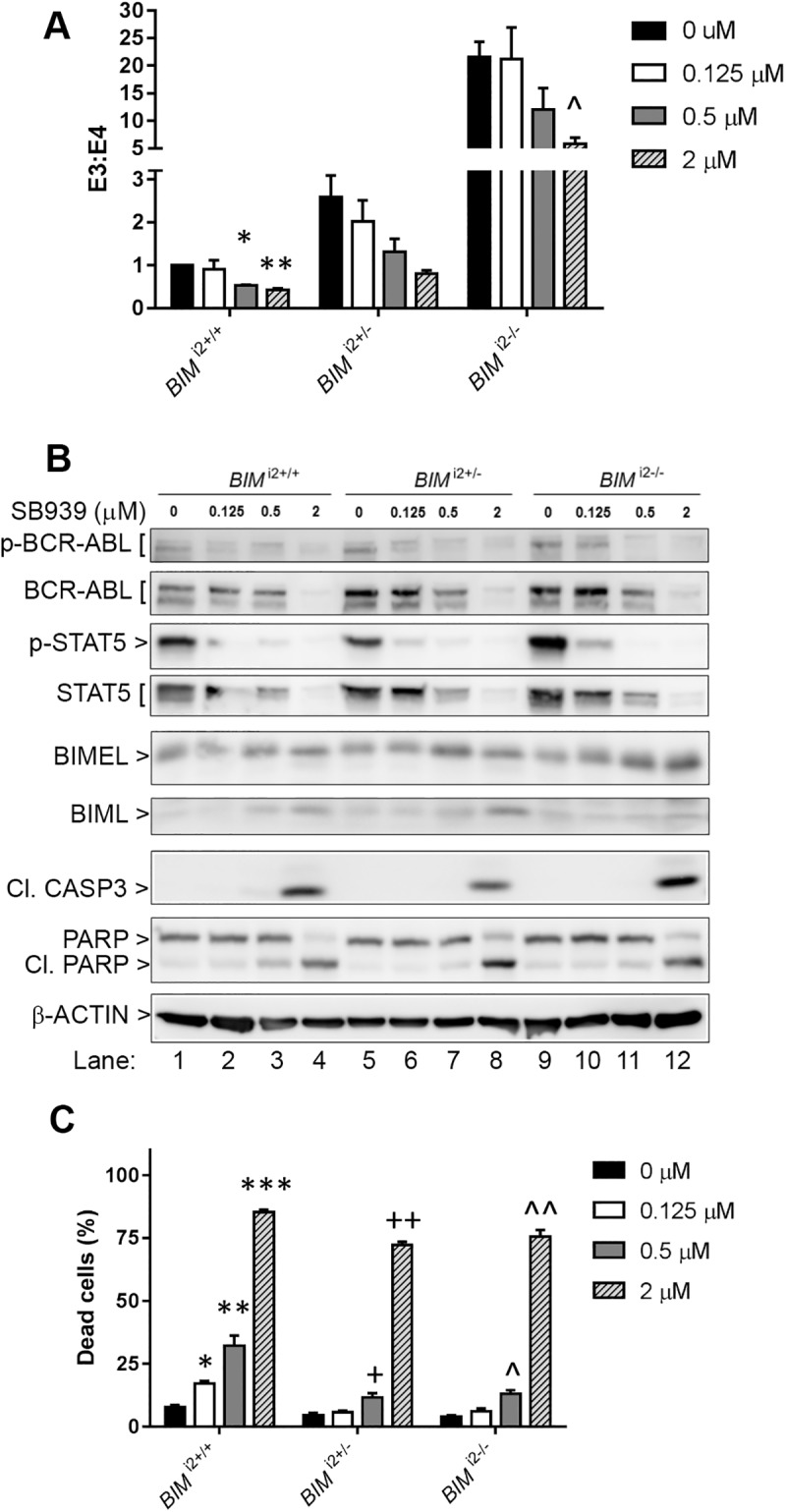
SB939 corrects pre-mRNA splicing of *BIM* and induces apoptosis in CML cell lines with the *BIM* deletion polymorphism. (A) Ratio of exon 3- to exon 4-containing *BIM* transcripts, as assayed by qPCR, in K562 CML cell lines without the *BIM* deletion polymorphism (*BIM*^*i2+/+*^), as well as those which were heterozygous (*BIM*^*i2+/-*^) and homozygous for the deletion polymorphism (*BIM*^*i2-/-*^). Cells were treated for 18 hours with SB939 at the indicated concentrations. (B) Immunoblots showing induction of apoptosis in K562 *BIM*^*i2+/+*^, *BIM*^*i2+/-*^ *and BIM*^*i2-/-*^. The data shown are representative of 3 experiments with similar results. Cells were treated for 48hours with SB939 at the indicated concentrations. p-BCR-ABL, phosphorylated BCR-ABL; STAT5, signal transducer and activator of transcription 5; p-STAT5, phosphorylated STAT5; BIMEL, BIM extra long isoform; BIML, BIM long isoform; Cl. CASP3, cleaved CASPASE3; Cl. PARP, cleaved PARP. 48-hour treatment as in A. (C) Apoptotic cell death, as assessed by flow cytometry-based Annexin V assay, in K562 *BIM*^*i2+/+*^, *BIM*^*i2+/-*^ *and BIM*^*i2-/-*^ cells after 48-hour treatment with SB939. All results are given as the mean ± s.e.m (n = 3). The P values were based on Student’s *t* test. For (A) and (C), the indicated P values were calculated by comparing to the corresponding DMSO control samples. (A): *P = 0.002, **P = 0.004, ^P = 0.019. (C): *P = 0.0031, **P = 0.02, ***P = 4.4 X 10^−7^, ^+^P = 0.037, ^++^P = 3.2 X 10^−6^, ^P = 0.0094, ^^P = 7.1 X 10^−4^.

Earlier reports have shown that vorinostat is effective in correcting splicing in epidermal growth factor receptor (EGFR)-mutated non-small cell lung cancer (NSCLC) cell lines harboring the *BIM* deletion polymorphism, and overcoming *BIM* deletion polymorphism-mediated EGFR TKI resistance [9]. Hence, we determined if SB939 would correct *BIM* splicing and overcome TKI resistance in CML cell lines with the *BIM* deletion polymorphism. First, we evaluated the effects of SB939 on isogenic K562 cells with the *BIM* deletion polymorphism in heterozygous (*BIM*^*i2+/-*^) and homozygous (*BIM*^*i2-/-*^) configurations, as well as control cells without the deletion polymorphism (*BIM*^*i2+/+*^) [5]. Consistent with our prior work, untreated *BIM* deletion polymorphism-containing K562 cells had increased E3/E4-containing *BIM* transcript ratios compared to control cells [5] (Fig 1A). We also found that treatment with SB939 decreased the E3/E4 transcript ratio in all three cell lines in a dose-dependent manner (Fig 1A). Consistent with the increase in E4-containing transcripts, SB939 exposure also increased protein expression of BH3-containing BIM isoforms, BIMEL and BIML (Fig 1B). The increase in the BIMEL and BIML protein isoforms was not associated with significantly increased cell death except at higher SB939 concentrations (2 uM) (Fig 1B). Interestingly, in addition to effects on BIM, SB939 at lower concentrations (0.125–0.5 uM) decreased BCR-ABL activity (as evidenced by decreased phosphorylation of BCR-ABL as well as its downstream target, STAT5 [13–15], while higher concentrations (2 uM) resulted in a decrease in both total BCR-ABL and STAT5 proteins (Fig 1B and S1 Fig). Lastly, we assayed apoptotic cell death using a flow cytometry-based Annexin V assay, and found that SB939 treatment increased cell death across all three cell lines in a dose-dependent manner, with the wildtype cells demonstrating more cell death except at the highest dose (2 uM) (Fig 1C).

### SB939 resensitizes *BIM* deletion polymorphism-containing CML cell lines to imatinib

**Fig 2:**
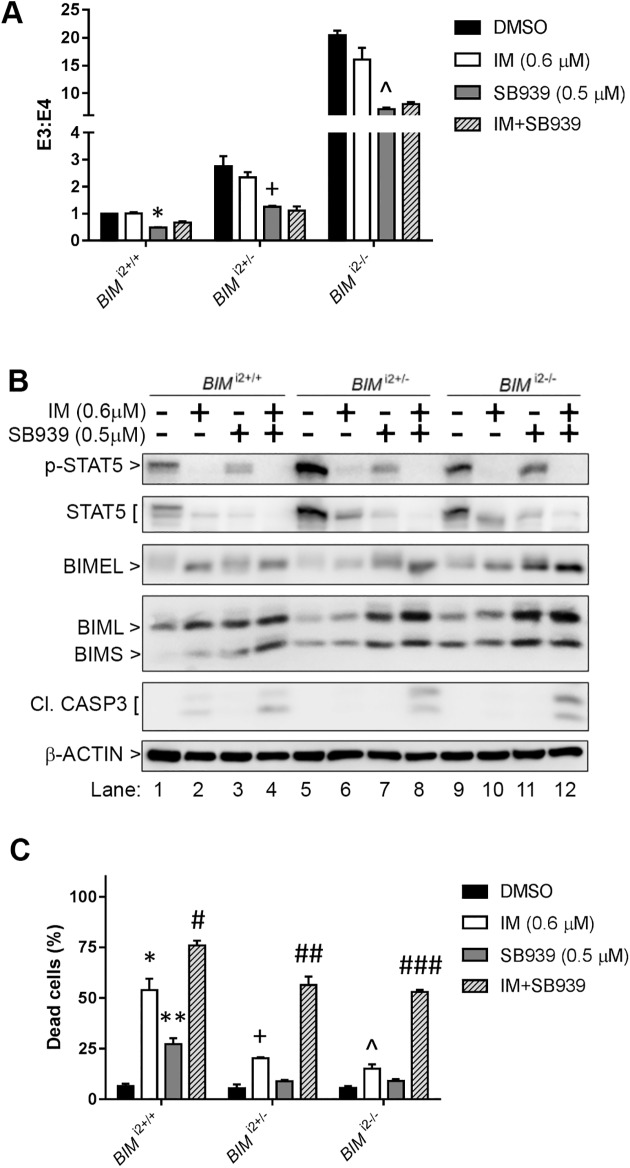
SB939 resensitizes *BIM* deletion polymorphism-containing CML cells to imatinib-induced apoptosis. (A) Ratio of exon 3- to exon 4-containing *BIM* transcripts, as measured by real-time qPCR, in K562 *BIM*^*i2+/+*^, *BIM*^*i2+/-*^ and *BIM*^*i2-/-*^ cells treated with 0.6 uM IM, 0.5 uM SB939, or both for 18 hours. (B) Immunoblots of cell lysates from K562 *BIM*^*i2+/+*^, *BIM*^*i2+/-*^ and *BIM*^*i2-/-*^ cells after 48 hours of treatment with the indicated drugs. The data shown are representative of 3 experiments with similar results. BIMS, BIM short isoform. (C) Apoptotic cell death, as assessed by a flow cytometry-based Annexin V assay, in K562 *BIM*^*i2+/+*^, *BIM*^*i2+/-*^ and *BIM*^*i2-/-*^ cells after 48-hour treatment with the indicated drugs. All results are given as the mean ± s.e.m (n = 3). The P values were based on Student’s *t* test. The following P values in (A) were calculated in comparison with samples treated with IM only: *P = 0.0058, ^+^P = 0.026, ^P = 0.047. For (C), the following P values were calculated by comparing with their respective DMSO-treated control samples: *P = 0.012, **P = 0.012, +P = 0.012, ^P = 0.028. The following P values were calculated in comparison to samples treated with IM only: ^#^P = 0.044 ^##^P = 0.012 ^###^P = 6.5 X 10^−4^.

Next, we determined the effects of combining SB939 and imatinib on *BIM* pre-mRNA splicing as well as *BIM* deletion polymorphism-induced TKI resistance. In all three K562 cell lines, we observed that SB939 consistently reduced the E3/E4 transcript ratio by approximately 50% but that imatinib had little effect, while the combination was as effective as SB939 alone (Fig 2A). Using Western blot, we observed that treatment with SB939 alone was able to induce higher levels of BIMEL, BIML, and BIMS expression in *BIM* deletion polymorphism-containing cells than imatinib alone (Fig 2B, compare lanes 6 to 7, and 10 to 11; S2 Fig). Importantly, whereas SB939 or imatinib alone was unable to induce caspase 3 cleavage in *BIM* deletion polymorphism-containing cells, the combination was able to do so (Fig 2B, compare lanes 8 to 6, and 12 to 10), thereby re-sensitizing *BIM* deletion polymorphism-containing cells to imatinib-induced apoptosis (Fig 2C).

**Fig 3:**
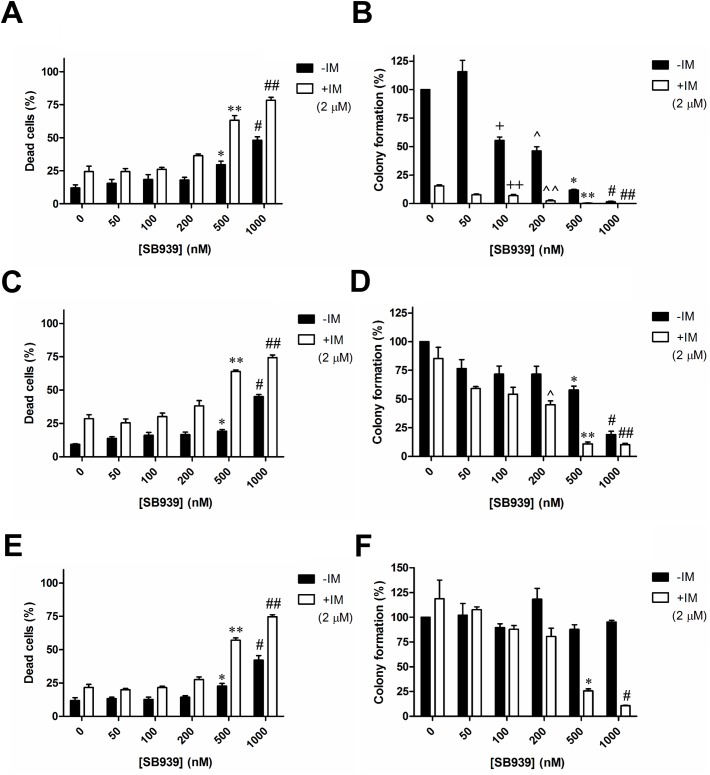
SB939, in combination with IM, significantly reduces the colony-forming ability of CML cell lines with the *BIM* deletion polymorphism. Results of apoptotic cell death, as assessed by flow cytometry-based Annexin V assay, for K562 (A) *BIM*^*i2+/+*^, (C) *BIM*^*i2+/-*^ and (E) *BIM*^*i2-/-*^ cells are shown. The P values were based on Student’s *t* test. Apoptotic cell death: *P = 0.0078 and ^#^P = 5 X 10^−4^ in (A), *P = 0.011 and ^#^P = 9.6 X 10^−4^ in (C), *P = 0.018 and ^#^P = 0.0033 in (E), were calculated by comparing with their respective DMSO-treated control samples. **P = 0.0022 and ^##^P = 0.0013 in (A), **P = 0.0032 and ^##^P = 4.8 X 10^−4^ in (C), **P = 3.3 X 10^−4^ and ^##^P = 10^−4^ in (E), were calculated in comparison with samples treated with IM only. Results of the colony formation assay for K562 (B) *BIM*^*i2+/+*^, (D) *BIM*^*i2+/-*^ and (F) *BIM*^*i2-/-*^ cells are shown. Colony formation for each sample was calculated as a percentage of the total number of colonies counted from the corresponding DMSO-treated control. The P values were based on Student’s *t* test. Colony formation assay: ^+^P = 0.0045, ^P = 0.0047, *P = 6 X 10^−5^ and ^#^P = 3 X 10^−5^ in (B), *P = 0.0059 and ^#^P = 0.0014 in (D), were calculated by comparing their respective colony formation to that of the DMSO controls. ^++^P = 0.0038, ^^P = 4.8 X 10^−4^, **P = 0.0026 and ^##^P = 0.0035 in (B), ^P = 0.041, **P = 0.014 and ^##^P = 0.015 in (D), *P = 0.038 and ^#^P = 0.029 in (F), were calculated by comparing their respective colony formation to that of the sample treated with IM only. Cells were treated with either or both SB939 (0-1000nM) and imatinib (2uM) for 72 hours. All results are given as the mean ± s.e.m (n = 3).

A detailed assessment of the effects of SB939, alone or in combination with IM, on the viability of these CML cell lines was performed (Fig 3). For all three cell lines, we observed that at least 500 nM SB939 induced significant levels of cell death and co-treatment with IM significantly increased levels of apoptosis as compared to their respective IM only treatment (Fig 3A, 3C and 3E). CFA showed that imatinib treatment alone dramatically reduced the number of colony in K562 *BIM*^*i2+/+*^ by approximately 80% but not those with the *BIM* deletion polymorphism (compare Fig 3B to 3D and 3F).When treated with SB939 alone, K562 *BIM*^i2+/+^ has the lowest IC_50_ (150nM) when compared to those with the *BIM* deletion polymorphism (*BIM*^i2+/-^, IC_50_ = 750nM; *BIM*^*i2-/-*^, IC_50_>1000nM; Fig 3B, 3D and 3F). However, the relatively high resistance to either SB939 or IM in cells with the *BIM* deletion polymorphism was overcome by combining SB939 and IM which resulted in a significant reduction of at least 5-fold in the IC_50_ of SB939 used (*BIM*^*i2+/-*^, IC_50_ = 150nM; *BIM*^*i2-/-*^, IC_50_ = 350nM; Fig 3D and 3F). Hence, the result strongly indicates synergy between SB939 and IM in significantly reducing the colony-forming ability of cells with the *BIM* deletion polymorphism.

### SB939 is effective against primary CML progenitors with the *BIM* deletion polymorphism

**Fig 4:**
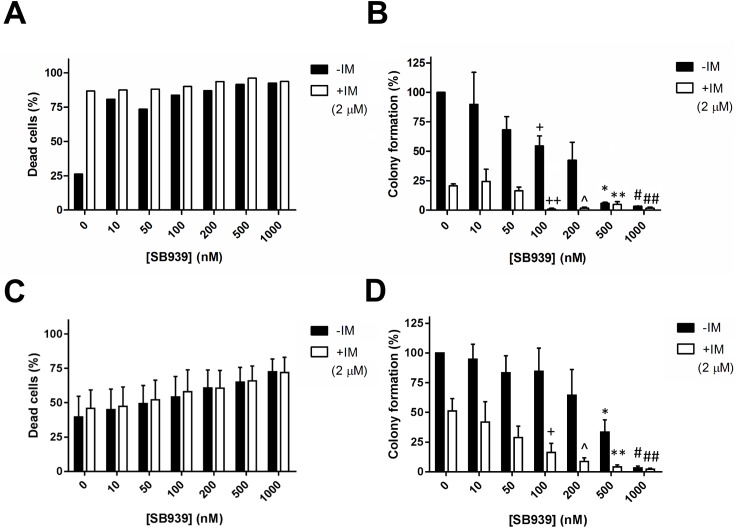
SB939 reduces the viability of primary CML progenitors with the *BIM* deletion polymorphism. Results of apoptotic cell death, as assessed by flow cytometry-based Annexin V assay, for primary CML cells (A) without (n = 3) and (C) with (n = 2) the *BIM* deletion polymorphism are shown. Results of the colony formation assay for primary CML cells (B) without (n = 3) and (D) with (n = 4) the *BIM* deletion polymorphism are shown. Colony formation for each sample was calculated as a percentage of the total number of colonies counted from the corresponding DMSO-treated control. The P values were based on Student’s *t* test. ^+^P = 0.033, *P = 10^−4^ and ^#^P = 2.8 X 10^−4^ in (B), *P = 0.0075 and ^#^P = 10^−6^ in (D) were calculated by comparing their respective colony formation to that of the DMSO controls. ^++^P = 0.0023, ^P = 0.0025, **P = 0.0071 and ^##^P = 0.0025 in (B), ^+^P = 0.038, ^P = 0.021, **P = 0.019 and ^##^P = 0.017 in (D) were calculated by comparing their respective colony formation to that of the sample treated with IM only. Cells were treated with either or both SB939 (0-1000nM) and imatinib (2uM) for 72 hours. All results are given as the mean ± s.e.m.

To determine if SB939 had activity against primary CML cells, we treated cells with or without the *BIM* deletion polymorphism with SB939, either alone or in combination with IM, for 72 hours (Fig 4). For primary CML cells without the *BIM* deletion polymorphism, we observed that at least 10 nM of SB939 was able to induce a 55% net apoptotic cell death, similar to that induced by 2 uM of IM, and that co-treatment with 2 uM IM did not significantly increase apoptotic cell death (Fig 4A). A parallel study was done on primary CML cells with the *BIM* deletion polymorphism (Fig 4C). Here, we observed that there was a dose-dependent increase in apoptosis, which 1000 nM SB939 induced 33% net apoptosis. However, the combination with IM did not increase the amount of apoptotic cell death significantly (Fig 4C). Colony formation assay was performed on primary CML cells without the *BIM* deletion polymorphism (Fig 4B). CFA showed that imatinib treatment alone dramatically reduced primary CML progenitors without the *BIM* deletion polymorphism by approximately 80% but only reduced those with the *BIM* deletion polymorphism by 50% (compare Fig 4B to 4D). When treated with SB939 alone, primary CML progenitors without the *BIM* deletion polymorphism has a lower IC_50_ (150nM; Fig 4B) when compared to that of those with the *BIM* deletion polymorphism (IC_50_ = 350nM; Fig 4D). However, the relatively higher resistance to either SB939 or IM in primary CML progenitors with the *BIM* deletion polymorphism was overcome by combining SB939 and IM which resulted in a significant reduction of at least 35-fold in the IC_50_ of SB939 used (single agent IC_50_ = 350nM; in combination, IC_50_ = 10nM; Fig 4D). Hence, there is a strong indication of synergy between SB939 and IM in significantly reducing the viability of the primary CML progenitors with the *BIM* deletion polymorphism.

## Discussion

The presence of the *BIM* deletion polymorphism can confer TKI resistance in CML by maintaining a higher ratio of anti-apoptotic exon 3- to pro-apoptotic exon 4-containing BIM transcripts [5]. SB939, however, can reverse the effect by reducing the ratio of exon 3- to exon 4-containing BIM transcripts (Fig 1A) which, translated to a significant increase in the induction of apoptosis in CML cells with the *BIM* deletion polymorphism (Fig 1B and 1C). Thus, SB939 corrects BIM pre-mRNA splicing in CML cell lines with the *BIM* deletion polymorphism in favour of apoptosis. We have previously shown that CML cells with the *BIM* deletion polymorphism were more resistant to imatinib when compared to their wildtype counterpart [5]. Unlike SB939, imatinib does not appear to change ratio of exon 3- to exon 4-containing BIM transcripts (Fig 2A) but it can increase the basal level of total BIM proteins which is further enhanced when combined with SB939 (Fig 2B). Furthermore, the observed inhibition of BCR-ABL activity by SB939 is consistent with previous reports on the ability of HDAC inhibitors to regulate the stability of BCR-ABL that is mediated by HDAC6 & HSP90 [16, 17]. Thus, SB939 in combination with imatinib can result in further enhancement of apoptosis in CML cell lines with the *BIM* deletion polymorphism (Figs 2B, 2C, 3C and 3E). Our findings are consistent with a previous report involving the HDAC inhibitor vorinostat and an EGFR TKI gefitinib on non-small cell lung cancer (NSCLC) with the *BIM* deletion polymorphism where vorinostat was also able to reduce the ratio of exon 3- to exon 4-containing BIM transcripts and thus, resensitized NSCLC cells with *BIM* deletion polymorphism to gefitinib [9]. CML patients with the *BIM* deletion polymorphism were found to respond poorly to standard dose of imatinib when compared to those without the deletion polymorphism [5]. SB939 alone can induce apoptosis in a dose-dependent manner in primary CML cells with the *BIM* deletion polymorphism (Fig 4C). More importantly, our colony-formation assays showed that SB939 decreases the viability of primary CML progenitors and in combination with IM, can further reduce the viability of primary CML progenitors especially in those with the *BIM* deletion polymorphism that are more resistant to both SB939 and IM (compare Fig 4B to 4D). In summary, our results indicate that SB939 overcomes the *BIM* deletion polymorphism-induced TKI resistance and SB939 should be considered as a therapeutic strategy for CML patients with TKI resistance associated with this deletion polymorphism.

## Supporting information

S1 Fig Immunoblot density ratios of the respective phosphorylated to total proteins for BCR-ABL and STAT5 related to Fig 1B. p-BCR-ABL, phosphorylated BCR-ABL; STAT5, signal transducer and activator of transcription 5; p-STAT5, phosphorylated STAT5.
(TIF)

S2 Fig Immunoblot density ratios of the three different protein isoforms of BIM to beta-ACTIN related to Fig 2B. BIMEL, BIM extra long isoform; BIML, BIM long isoform; BIMS, BIM short isoform.
(TIF)
