# Supplementary figures and images for: The HDAC inhibitor SB939 overcomes resistance to BCR-ABL kinase Inhibitors conferred by the BIM deletion polymorphism in chronic myeloid leukemia
Source: PLoS One. 2017 Mar 16;12(3):e0174107. doi: 10.1371/journal.pone.0174107 (PMC5354438; doi:10.1371/journal.pone.0174107)

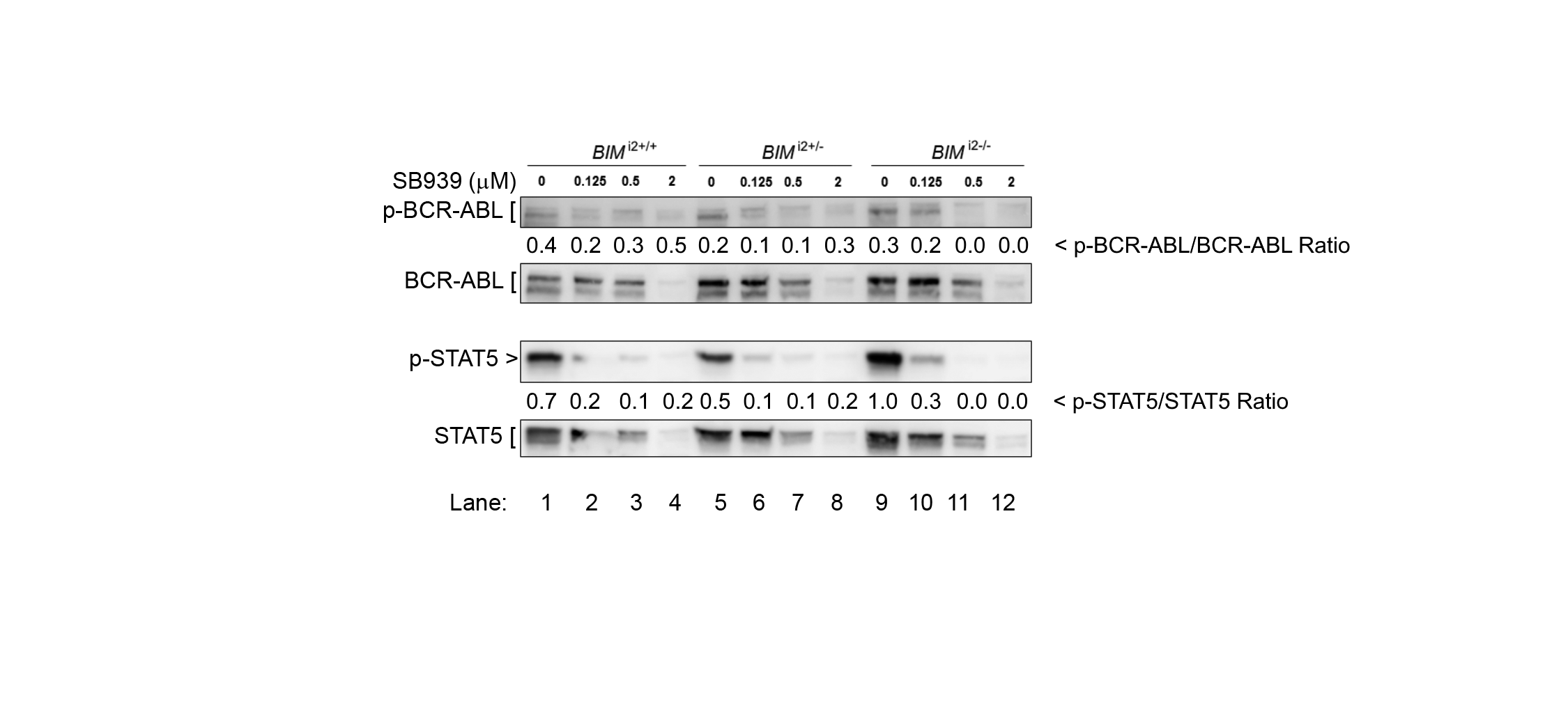

Supplement: S1 Fig — p-BCR-ABL, phosphorylated BCR-ABL; STAT5, signal transducer and activator of transcription 5; p-STAT5, phosphorylated STAT5. (TIF) [file pone.0174107.s001.tif]

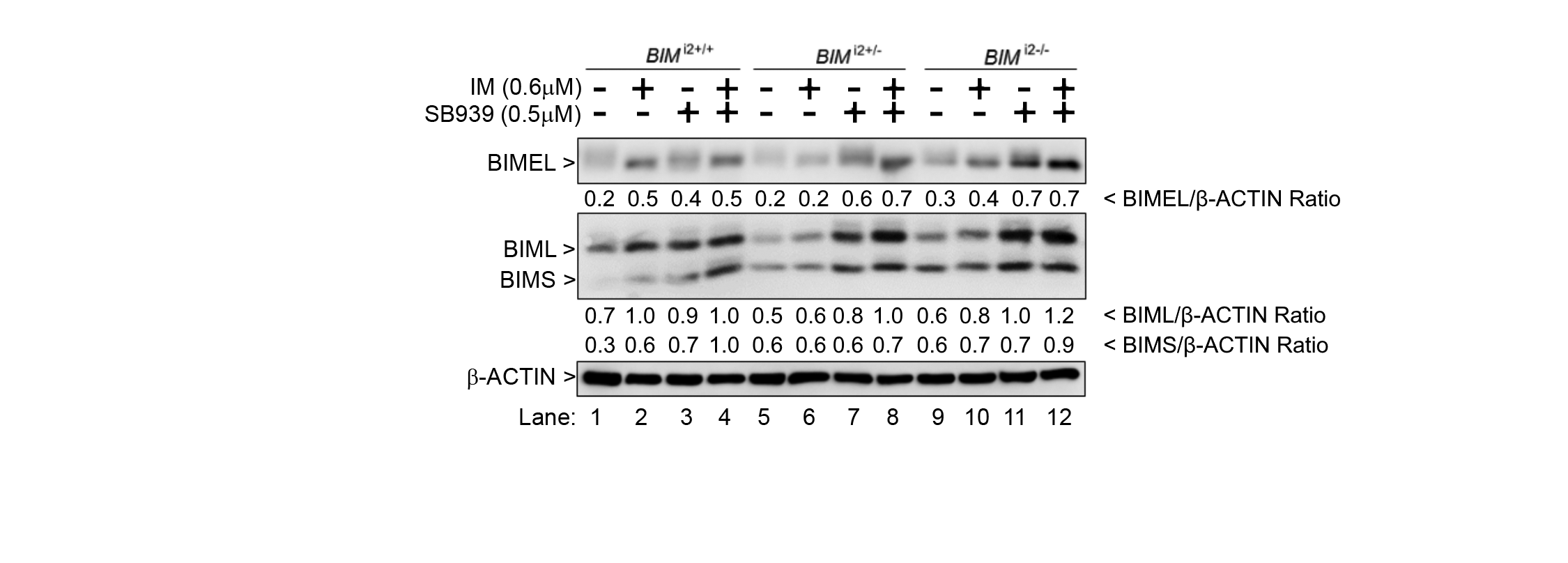

Supplement: S2 Fig — BIMEL, BIM extra long isoform; BIML, BIM long isoform; BIMS, BIM short isoform. (TIF) [file pone.0174107.s002.tif]
